# CEACAM6 serves as a biomarker for leptomeningeal metastasis in lung adenocarcinoma

**DOI:** 10.1002/cam4.5221

**Published:** 2022-09-09

**Authors:** Xueying Wang, Xuemei Tang, Jiahui Gu, Ziwei Sun, Shengrui Yang, Yuan Mu, Ming Guan, Kun Chen, Wei Liu, Haoyu Ruan, Jian Xu

**Affiliations:** ^1^ Department of Laboratory Medicine The First Affiliated Hospital of Nanjing Medical University Nanjing China; ^2^ Central Laboratory Huashan Hospital, Fudan University Shanghai China; ^3^ Branch of National Clinical Research Center for Laboratory Medicine Nanjing China; ^4^ Department of Laboratory Medicine Huashan Hospital, Fudan University Shanghai China; ^5^ School of Internet of Things Engineering, Wuxi University Wuxi China

**Keywords:** biomarker, CEACAM6, leptomeningeal metastasis, lung adenocarcinoma

## Abstract

**Background and aims:**

Diagnosis of leptomeningeal metastasis (LM) is challenging. In our previous study, CEACAM6 mRNA was found to be highly expressed in the circulating tumor cells of cerebrospinal fluid (CSF) from patients with lung adenocarcinoma with LM (LUAD‐LM). The aim of this study was to identify whether CEACAM6 could be used as a biomarker for LUAD‐LM.

**Materials and methods:**

The level of CEACAM6 was determined by enzyme‐linked immunosorbent assay (ELISA) in CSF from 40 LUAD‐LM and 44 normal controls, and additional serum samples from 138 LUAD patients, including 12 LUAD‐LM patients, and 30 healthy controls. Carcinoembryonic antigen (CEA), cytokeratin 19 fragment (CYFRA 21‐1) and neuron‐specific enolase (NSE) levels in the CSF and sera were detected by chemiluminescent immunoassay. Receiver operating characteristic curve was plotted to evaluate the diagnostic performance for LUAD‐LM.

**Results:**

CSF CEACAM6 level was higher in LUAD‐LM than that in normal controls. In serum, LUAD patients had a higher level of CAECAM6 than healthy controls, and LM patients had the highest level among them. Serum CEACAM6 had a higher AUC than CEA in differentiating LM from non‐LM in LUAD patients (0.95 vs. 0.64, *p* < 0.001).

**Conclusion:**

CEACAM6 may serve as a potential biomarker in diagnosing LUAD‐LM.

## Graphical abstract

CEACAM6 in the CSF was higher in LUAD‐LM than benign diseases. LUAD‐LM patients had a higher level of serum CAECAM6 than healthy controls and non‐LM LUAD patients. Serum CEACAM6 had a higher AUC than CEA in differentiating LM from non‐LM in LUAD. CEACAM6 may serve as a potential biomarker in diagnosing LUAD‐LM.

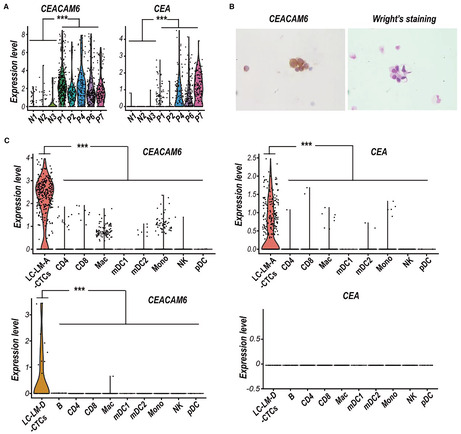

## 1 INTRODUCTION

Leptomeningeal metastasis (LM) occurs in approximately 3%–5% of patients with advanced non‐small‐cell lung cancer (NSCLC).^1^ Lung adenocarcinoma (LUAD) is the most frequent subtype of NSCLC suffering from LM. The median survival of patients with lung adenocarcinoma leptomeningeal metastasis (LUAD‐LM) remains 3–11 months.^2^, ^3^ Diagnosis of LM is challenging, and depends on clinical presentation, CSF cytology and imaging. The diagnostic gold standard is a positive CSF cytology. However, cancer cells in the CSF are scarce, which is the major limitation of the diagnosis. A retrospective study enrolled 334 patients with advanced lung cancer, 22% were diagnosed with LM by CSF cytology alone, 35% by magnetic resonance imaging (MRI) alone, and 42% by combining both.^4^ Novel biomarkers and detection approaches for LM diagnosis are required urgently.

Based on single cell RNA sequencing, our previous study discovered CSF‐CTCs of five LUAD‐LM patients had a high expression of CEACAM6,^5^ which was also defined in CSF tumor cells from four NSCLC‐LM patients.^6^ Interestingly, CEACAM6 expression was higher than traditional biomarker CEACAM5 (CEA) in CSF tumor cells. The high expression of CEACAM6 in CSF tumor cells indicates its potential in the LUAD‐LM diagnosis.

The carcinoembryonic antigen‐related adhesion molecules (CEACAMs) are a large family, among which CEACAM5 (CEA) and CEACAM6 are recognized as related to cancer processes.^7^ CEA is a commonly used tumor marker, which is abnormally expressed in colorectal cancer, pancreatic cancer, breast cancer and LUAD.^8^ CEA exhibits one variable (V)‐like domain, identified as the N domain, followed by six constant C2‐like Ig domains. Whereas the structure of CEACAM6 protein was a N domain followed by two C2‐like domains.^7^ The structure differences contribute to the diversity of functions ascribed to the two molecules.^7^ Overexpression of CEACAM6 promotes cancer progression through aberrant cell differentiation, anti‐apoptosis, cell growth and epithelial‐mesenchymal transition (EMT).^9^, ^10^ Son et al. reported that CEACAM6 gene silencing showed therapeutic potential in LM‐LUAD.^11^

In this study, we intend to analyze the CSF and serum CEACAM6 level in LUAD‐LM patients, compared to traditional biomarkers like CEA, cytokeratin 19 fragment (CYFRA 21‐1) and neuron‐specific enolase (NSE). We found that the CEACAM6 level was high in the CSF and serum of LUAD ‐LM patients. The area under the curve (AUC) of serum CEACAM6 for the diagnosis of LUAD‐LM was higher than that of CEA, CYFRA 21‐1 and NSE. CEACAM6 may serve as a potential biomarker for LUAD‐LM.

## 2 METHODS AND MATERIALS

### 2.1 Patients and samples

CSF samples were collected from 40 LUAD‐LM patients and 44 controls at Huashan Hospital between July and December 2021. The control group consisted of benign disease patients with normal results of routine CSF analysis and cytology.

Serum samples came from 138 LUAD patients, including 12 LM cases, and 30 healthy controls (HC) from January 2020 to December 2021, which were obtained from the blood sample bank of the First Affiliated Hospital of Nanjing Medical University. The study workflow is provided in Figure S1.

All LUAD patients were pathologically confirmed. Clinicopathological factors and clinical stage were classified according to the 8th edition of the Union for International Cancer Control (UICC) tumor‐node‐metastasis (TMN) system. LM patients were diagnosed based on positive CSF cytology results. Clinical data, including patient age, gender, clinical and histological grade, CEA, CYFRA 21‐1 and NSE level were recorded. Serum samples were separated from whole blood after centrifugation (3000 rpm, 5 min) and stored at −80°C, waiting for ELISA detection. This research was approved by the Research and Ethical Committee of the First Affiliated Hospital of Nanjing Medical University (2022‐SRFA‐037).

### 2.2 Sample testing

CSF and serum samples were collected, and the level of CEACAM6 were detected by ELISA (Sino Biological) according to the instruction of the manufacturer. The level of CEA, CYFRA 21‐1 and NSE in CSF and serum were detected by Chemiluminescent Immunoassay (CLIA) by using Roche Cobas e602 according to the instruction of the manufacturer.

### 2.3 Statistical analyses

Statistical analyses were performed with SPSS 22.0 (IBM Corp) and GraphPad Prism Software (GraphPad Inc). *p* < 0.05 was considered statistically significant. All the data were presented in median and interquartile range (IQR). The distribution of serum biomarkers was skewed. Nonparametric tests such as the Wilcoxon rank‐sum test or Kruskal–Wallis test were used for continuous variables. In order to assess the diagnostic efficiency of CEACAM6, receiver‐operating characteristic (ROC) curves were plotted. By ROC curve analysis, the area under the curve (AUC) including 95% confidence interval (CI) values were determined.

## 3 RESULTS

### 3.1 Single‐cell analysis map reveals that *CEACAM6* mRNA increased in CSF of LUAD‐LM patients

**FIGURE 1:**
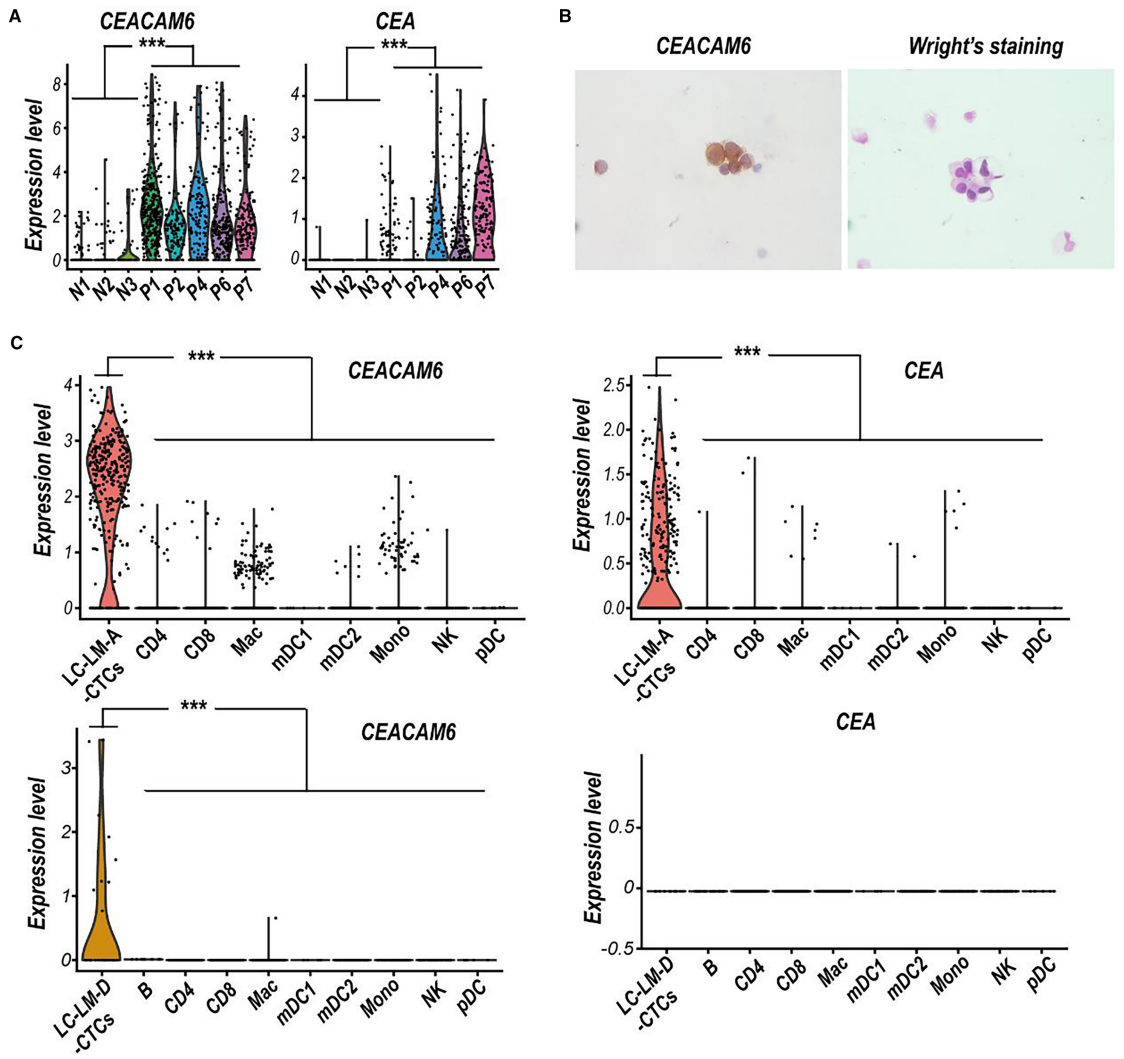
scRNA‐seq analysis reveals that *CEACAM6* mRNA increased in CSF of LUAD‐LM patients. (A) Violin plots of *CEACAM6* and *CEA* mRNA expression in cells of three normal CSF samples (N1, N2 and N3) and cerebrospinal fluid circulating tumor cells (CSF‐CTCs) of five LUAD‐LM patients CSF samples (P1, P2, P4, P6 and P7). ***p‐value <0.001, Wilcoxon rank‐sum test. (B) The CEACAM6 immunocytochemistry and Wright's staining of CSF cells of a LUAD‐LM patient. (C) Violin plots of *CEACAM6* and *CEA* mRNA expression in CSF‐CTCs and various immune cells of LUAD‐LM patient A (LUAD‐LM‐A, up) or LUAD‐LM patient D (LUAD ‐LM‐D, down) CSF samples. Cluster key: pDC, plasmacytoid dendritic cells; mDC1, myeloid DC type 1; mDC2, myeloid DC type 2; Mono, monocytes; Mac, macrophages; CD8, CD8+ T cells; CD4, CD4+ T cells; NK, natural killer cells; B, B cells.

Our previous single‐cell RNA sequencing data of CSF circulating tumor cell (CSF‐CTCs) from five LUAD‐LM patients indicated that *CEACAM6* mRNA was significantly upregulated compared to normal CSF cells (Figure 1A), and the immunohistochemistry staining also showed high expression of CEACAM6 in CSF‐CTCs (Figure 1B). Based on 10× genomics scRNA‐seq data of two LUAD‐LM patients from the GEO database (LUAD‐LM‐A, GSM4555887; and LUAD‐LM‐D, GSM4555890), we further confirmed the high levels of *CEACAM6* expression in CSF‐CTCs compared to various CSF immune cells (Figure 1C). Although the higher *CEA* mRNA expression were also detected in CSF‐CTCs of LUAD‐LM patients compared to non‐malignant cells, the expression level of *CEA* was lower than *CEACAM6* in CSF‐CTCs (Figure 1C). CEACAM6 had a high possibility to serve as a diagnostic biomarker for LUAD‐LM, which made it more reliable and potent than CEA.

### 3.2 CEACAM6 increased in CSF of LUAD‐LM patients

**FIGURE 2:**
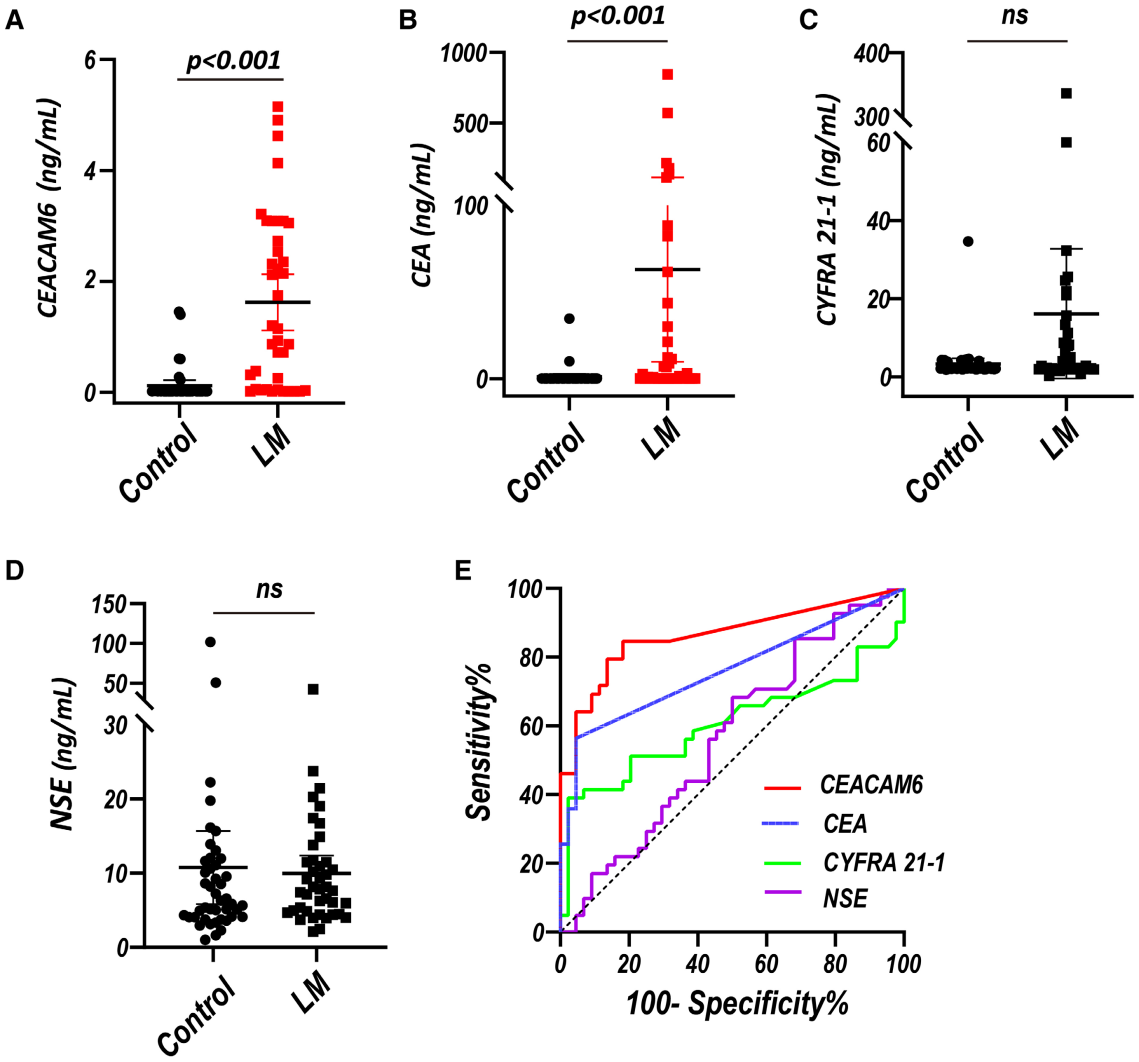
Levels of CSF CEACAM6 in LUAD‐LM patients. (A–D) The level of CSF CEACAM6, CEA, CYFRA 21‐1 and NSE in LUAD‐LM patients (LM) and normal controls. (E) ROC curve of CSF CEACAM6 in differentiating between LM and controls.

To verify the results from scRNA‐seq, we performed ELISA for the levels of CEACAM6 in the CSF from 40 LUAD‐LM patients (male: 42.5%, median age of 57.0 years with IQR of 50.0–65.8 years) and 44 controls with normal results of routine CSF examination and cytology (male: 52.3%, median age of 50.0 years with IQR of 37.0–58.8 years) (Table S1). CSF CEACAM6 could be detected in 34/40 LM (median value of 2.130 ng/ml with IQR of 0.633–3.088 ng/ml) and 14/44 controls (median value of 0.039 ng/ml with IQR of 0.024–0.600 ng/ml). While CSF CEA could be detected in 22/40 LM (median value of 25.63 ng/ml with IQR of 5.84–120.71 ng/ml) and in 2/44 controls (10.10 and 34.64 ng/ml). The level of CSF CEACAM6 and CEA in LM were both higher than those in controls (both *p* < 0.001, Figure 2A,B **)**, whereas CSF CYFRA21‐1 and NSE level showed no statistical difference between LM and controls (both *p* > 0.05, Figure 2C,D).

By setting an optimum cutoff value of 0.031 ng/ml, CSF CEACAM6 had a sensitivity of 85.0% and a specificity of 81.8% with an AUC of 0.87 (95% CI: 0.79–0.95) for discriminating LUAD‐LM from controls. Whereas CSF CEA had a sensitivity of 56.4% and a specificity of 95.5% with an AUC of 0.75 (95% CI: 0.65–0.86) by setting an optimum cutoff value of 50.54 ng/ml (Figure 2E) (Table 1). In addition, the combination of CSF CEACAM6 and CEA achieved an AUC of 0.89 (95%CI: 0.82–0.96), which showed no significant increase compared with CEACAM6 alone (Figure S2) (Table 1). However, the AUC of CSF CYFRA 21‐1 and NSE was only 0.62 (95% CI: 0.49–0.75) and 0.56 (95% CI: 0.44–0.69) respectively (Figure 2E) (Table 1). The discrimination efficacy of CEACAM6 was better than that of CEA, CYFRA 21‐1 and NSE in the CSF.

**TABLE 1:** Diagnostic performance of CEACAM6 in CSF for LUAD‐LM

	AUC	95% CI	Sensitivity (%)	Specificity (%)	PPV (%)	NPV (%)
CEACAM6	0.87	0.79–0.95	85.0	81.8	81.0	85.7
CEA	0.75	0.65–0.86	56.4	95.5	91.9	70.7
CYFRA 21‐1	0.62	0.49–0.75	40.0	97.7	94.1	64.2
NSE	0.56	0.44–0.69	67.5	50.0	55.1	62.9
CEACAM6 + CEA	0.89	0.82–0.96	87.5	81.8	81.4	87.8

### 3.3 CEACAM6 increased in the Sera of LUAD‐LM patients

**FIGURE 3:**
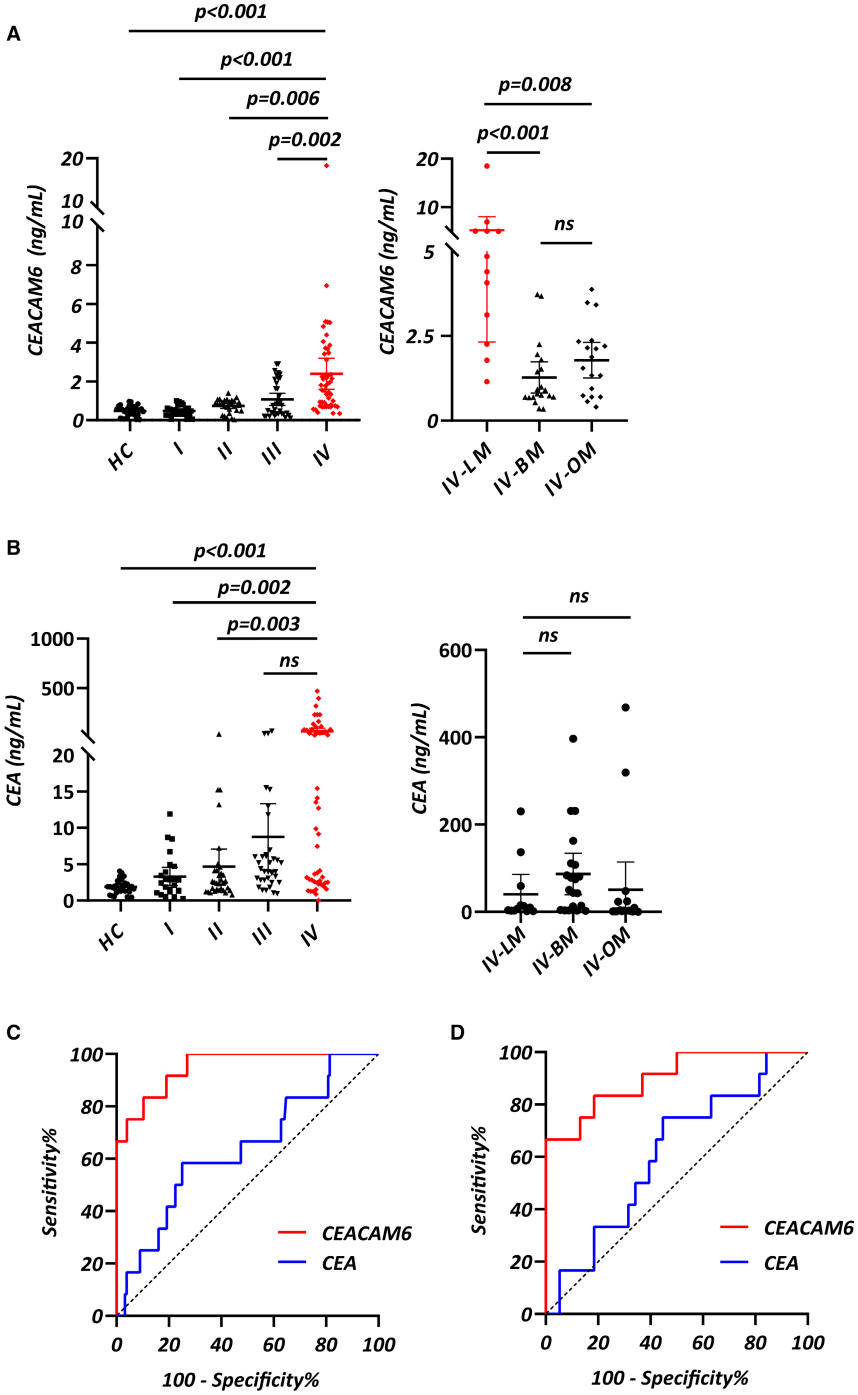
Levels of serum CEACAM6 in LUAD patients. (A, B) The level of serum CEACAM6 (A) and CEA (B) in LUAD patients and healthy individuals (HC). Patients in stage IV were further divided into leptomeningeal metastasis (LM), brain metastasis (BM) and other organ metastasis (OM) which included bone, liver, kidney, pleura, pericardium and adrenal gland metastases. (C, D) ROC curves of serum CEACAM6 and CEA for LM diagnosis both in the LUAD patients (C) and stage IV LUAD patients (D).

Furtherly, sera CEACAM6 levels in 30 HC individuals (male: 50.0%, median age of 45.0 years with IQR of 38.8–51.0 years) and 138 LUAD patients (Stage I: male: 50.0%, median age of 54.0 years with IQR of 41.3–63.0 years; Stage II: male: 62.1%, median age of 58.0 years with IQR of 49.0–68.5 years; Stage III: male: 72.2%, median age of 63.0 years with IQR of 55.0–69.0 years; Stage IV: male: 44.0%, median age of 59.0 years with IQR of 52.8–68.3 years) were detected (Table S2). Median values of serum CEACAM6 in HC and LUAD patients from stage I to stage IV were 0.491 (0.275, 0.697) ng/ml, 0.467 (0.305, 0.627) ng/ml, 0.812 (0.501, 1.01) ng/ml, 0.754 (0.325, 2.122) ng/ml, 1.66 (0.73, 3.42) ng/ml, respectively. The level of serum CEACAM6 was higher in LUAD patients than in HC, and it showed an increasing trend from stage I to stage IV (Figure 3A). Although the serum CEA level was higher in LUAD patients than in HC, however, there was no statistical difference between stage IV and stage III (Figure 3B). In order to further analyze the level of CEACAM6 in metastatic LUAD patients, we divided stage IV patients into 3 subgroups including leptomeningeal metastasis (LM, male: 50.0%, median age of 57.5 years with IQR of 54.3–61.8 years), brain metastasis (BM, male: 40.0%, median age of 55.5 years with IQR of 50.5–66.5 years) and other organ metastasis (OM, male: 44.4%, median age of 65.5 years with IQR of 54.3–69.5 years). LM patients had higher serum CEACAM6 level than both BM and OM patients, whereas no statistical difference was found between BM and OM subgroups **(**Figure 3A **)**. However, there was no significant difference in serum level of CEA among LM, BM and OM patients (Figure 3B). Additionally, serum CYFRA 21‐1 and NSE level showed significant difference neither among different stages of LUAD nor among LM, BM and OM subgroups (Figure S3).

The AUC of serum CEACAM6 was 0.95 (95% CI: 0.90–1.00) for diagnosing LM in LUAD patients, which was larger than that of serum CEA (AUC = 0.64, 95% CI: 0.47–0.80, *p* < 0.001).

The cutoff value was 1.119 ng/ml for serum CEACAM6 and 7.34 ng/ml for serum CEA, with a sensitivity and a specificity of 100.0% and 73.0%, 58.3% and 75.0% respectively (Figure 3C) (Table 2). For differentiating between LM and non‐LM in stage IV patients, serum CEACAM6 had sensitivity of 66.7% and specificity of 100.0% by setting a cutoff value of 3.967 ng/ml. Meanwhile, serum CEA had sensitivity of 75.0% and specificity of 45.5% by setting a cutoff value of 19.24 ng/ml. The diagnostic efficiency of CEACAM6 was superior to CEA in serum (CEACAM6: AUC = 0.90, 95% CI = 0.80–1.00; CEA: AUC = 0.53, 95% CI = 0.35–0.70; *p* < 0.001) (Figure 3D) (Table 2). The AUC of serum CYFRA21‐1 was 0.51 (95% CI: 0.31–0.71) in discriminating LM from LUAD patients, while the AUC of serum NSE was 0.56 (95% CI: 0.39–0.73). A cutoff value was 11.11 ng/ml for serum CYFRA21‐1 and 14.15 ng/ml for serum NSE, with a sensitivity and a specificity of 25.0% and 96.8%, 83.3% and 35.7% respectively (Figure S3) (Table 2). For differentiating between LM and non‐LM patients of stage IV, serum CYFRA21‐1 with a cut off value of 10.98 ng/ml had an AUC of 0.51 (95% CI: 0.32–0.71, sensitivity: 25.0%, specificity: 92.1%). Serum NSE with a cut off value of 14.74 ng/ml had an AUC of 0.53 (95% CI: 0.35–0.72, sensitivity: 75.0%, specificity: 44.7%) (Figure S3) (Table 2). These results indicated that the sera level of CEACAM6 were elevated especially in LUAD‐LM patients. Compared with CEA, CYFRA21‐1 and NSE, serum CEACAM6 showed better diagnostic performance for LUAD‐LM.

**TABLE 2:** Diagnostic performance of CEACAM6 in serum for LUAD‐LM

	AUC	95% CI	Sensitivity (%)	Specificity (%)	PPV (%)	NPV (%)
Stage I–IV						
CEACAM6	0.95	0.90–1.00	100.0	73.0	77.1	100.0
CEA	0.64	0.47–0.80	58.3	75.0	67.9	66.4
CYFRA 21‐1	0.51	0.31–0.71	25.0	96.8	87.8	58.7
NSE	0.56	0.39–0.73	83.3	35.7	54.1	70.2
Stage IV						
CEACAM6	0.90	0.80–1.00	66.7	100.0	100.0	76.7
CEA	0.53	0.35–0.70	75.0	45.5	60.4	71.0
CYFRA 21‐1	0.51	0.32–0.71	25.0	92.1	74.2	54.5
NSE	0.53	0.35–0.72	75.0	44.7	55.2	66.3

## 4 DISCUSSION

LUAD patients with LM have increased significantly due to improved therapy and prolonged survival. However, the survival of LUAD‐LM is still very poor. Particularly, LUAD patients with EGFR mutations were much more likely to suffer from LM.^12^, ^13^ The diagnosis of LM is mainly based on CSF cytology and MRI. However, the highest sensitivity for traditional diagnostic approaches is only approximately 70%, and correct interpretation of results is highly dependent on the skill and experience of the pathologists or the radiologists.

Novel approaches which might improve the diagnosis of LM are being investigated. Several studies showed that evaluation of CSF circulating tumor DNA (ctDNA) had the potential to facilitate the diagnosis of LM.^14^, ^15^, ^16^ Droplet digital PCR (ddPCR) was often employed to detect EGFR mutation in the CSF of NSCLC‐LM patients.^17^ Because of the advantage of gene amplification, ctDNA detection is more sensitive than CSF cytology. In a study of NSCLC‐LM, EGFR mutation in CSF ctDNA was identified in all seven cases, whereas CSF cytology was positive in only two of seven patients.^18^ However, its wide adoption is currently hampered by technical complexity and high cost. Novel applicable biomarkers of LM for clinical use are urgently required.

In this article, based on single‐cell RNA sequencing data, we found that CEACAM6 mRNA were upregulated in CSF cancer cells from LUAD‐LM patients. Moreover, the mRNA level of *CEACAM6* was higher than *CEA* in CSF‐CTCs, which suggested that CEACAM6 might conferred LUAD cells with enhanced metastatic ability and meningophilicity. A recent study also demonstrated that tumor‐associated CEACAM6 cfRNA was present in CSF samples from NSCLC‐LM patients.^6^ In addition, Kobayashi et al. firstly reported that CEACAM6 was highly expressed in LUAD tissues and correlated with clinical outcomes.^19^ Singer et al. reported that CEACAM6 was upregulated in LUAD cell lines and promoted cell growth.^9^ Proteomic analysis identified that CEACAM6 could distinguish benign pulmonary nodules from LUAD.^20^ Overexpression of CEACAM6 could activate Src‐FAK signaling and inhibits anoikis in LUAD.^21^ Anti‐CEACAM6 monoclonal antibody could enhance anoikis sensitivity and inhibit metastasis in LUAD, which suggested that anoikis resistance induced by CEACAM6 facilitates tumor cells metastasis and survival in the CSF.^22^, ^23^ Although the important roles of CEACAM6 in the cancer metastasis have been conformed in various studies, the exact molecular mechanism underlying the promoting effect of CEACAM6 overexpression on LM is still unclear for the difficulty on the construction of a leptomeningeal metastasis model.

We further detected CEACAM6 protein level in CSF and serum by ELISA. As expected, CEACAM6 protein level was significantly upregulated in the CSF of LUAD‐LM patients compared to normal controls. Moreover, the detection frequency of CEACAM6 was much higher than CEA in the CSF of LUAD‐LM patients. This result was consistent with our single‐cell RNA sequencing study.^5^ Previous study has investigated the diagnostic values of several biomarkers including β‐glucuronidase, LDH and β2‐microglobulin. Although the levels of these biomarkers were upregulated in the CSF of LM patients, they were also elevated in some benign diseases such as infections.^24^ The specificity of CSF CEACAM6 for LUAD‐LM patients suggested that this molecule was an oncogenic protein, but not a tumor responsive protein.

We also found that serum CEACAM6 was increased in LUAD patients with tumor progression and reached the peak in LM. To evaluate the diagnostic capability of serum CEACAM6 for LUAD‐LM, ROC curve analysis was performed. It revealed that serum CEACAM6 could not only discriminate LM from early stage LUAD patients, but also from advanced LUAD. Compared with traditional LUAD biomarkers, CEACAM6 showed a decent diagnostic performance for LUAD‐LM. CEA is a classical tumor marker, which is widely used in the auxiliary diagnosis and monitoring in several malignancies. The elevation of serum CEA level often suggests tumor progression or recurrence. The very high level of serum CEA always indicates metastasis.^25^ We detected a high amount of CEA in the serum of LUAD patients with metastasis. However, no difference was found between LM and other types of metastases. It suggested that CEA was a biomarker for LUAD, but not for LUAD‐LM.

Additionally, serum CEACAM6 was much higher in LM than other metastases such as brain metastasis and bone metastasis. It suggested that LUAD cells with high CEACAM6 expression were more prone to LM. Integrins are transmembrane receptors which have been implicated in cell adhesion and migration.^26^ Integrin determines organotrophic metastasis of cancer cells.^27^ Integrins α6β4 and α6β1 were associated with lung metastasis, while integrin αvβ5 was linked to liver metastasis.^28^ Integrin αvβ3 could promote efficient brain metastasis.^29^ CEACAM6 could enhance the binding between integrin αvβ3 on pancreatic cancer cells and the extracellular matrix,^30^ and promote integrin α5β1 on the colorectal cancer cells to bind to the fibronectin receptors of fibroblast cells.^31^ Whether CEACAM6 promotes LM of LUAD by increasing the interaction between integrin and leptomeninges is worth studying.

Compared with traditional methods, serum CEACAM6 measurement has some advantages in the diagnosis of LUAD‐LM. First, serum CEACAM6 had high sensitivity and specificity for LUAD‐LM diagnosis. Second, the detection of serum CEACAM6 is noninvasive and easy for patients to accept. Dynamic monitoring of serum CEACAM6 will facilitate early detection of LM in LUAD patients. Finally, because both CEACAM6 measurement and result interpretation are simple, it is easy to be widely used in clinical practice.

This research had some limitations. First, for lung cancer patients with cerebral symptoms, if no MRI evidence of brain metastases were discovered, very few patients would perform the CSF examination. Therefore, it is very difficult to obtain CSF samples from lung cancer patients without LM, and we did not collect such specimens for detecting the level of CSF CEACAM6 in this study. Due to the lack of CSF samples of LUAD patients without LM, the evidence in this work is not strong enough to fully prove that CEACAM6 could be used as biomarker to distinguish LUAD‐LM from LUAD patients. Second, the sample size of sera from LUAD‐LM was relatively small. Finally, because there are no in vitro diagnostics products for CEACAM6 detection in clinical practice at present, the assay for CEACAM6 quantification in this work was not a clinically approved method. Further studies need to add CSF samples of LUAD patients without LM and enlarge the sample size to confirm the diagnostic value of CEACAM6.

In conclusion, this study suggests that CEACAM6 is a potential biomarker for LM in LUAD. The detection of CEACAM6 may be helpful for timely diagnosis and monitoring of LUAD‐LM, thus improving the prognosis.

## AUTHOR CONTRIBUTIONS

Jian Xu and Haoyu Ruan designed the study; Xueying Wang, Xuemei Tang, Jiahui Gu and Ziwei Sun performed the experiments; Xueying Wang, Xuemei Tang, Shengrui Yang and Kun Chen collected clinical data and samples; Xueying Wang, Xuemei Tang, Yuan Mu and Wei Liu performed statistical analysis; Jian Xu, Haoyu Ruan, Xueying Wang and Xuemei Tang wrote the manuscript with help from all authors; Ming Guan supported and coordinated the research.

## CONFLICT OF INTEREST

Authors state no conflict of interest.

## ETHICS APPROVAL AND CONSENT TO PARTICIPATE

This research was approved by the Research and Ethical Committee of the First Affiliated Hospital of Nanjing Medical University.

## Supporting information


Figure S1

Figure S2

Figure S3

Table S1
Table S2

## Data Availability

The data that support the findings of this study are available from the corresponding authors upon reasonable request.

## References

[1] Cheng H , Perez‐Soler R . Leptomeningeal metastases in non‐small‐cell lung cancer. Lancet Oncol. 2018;19(1):e43‐e55. 29304362 10.1016/S1470-2045(17)30689-7

[2] Remon J , Le Rhun E , Besse B . Leptomeningeal carcinomatosis in non‐small cell lung cancer patients: a continuing challenge in the personalized treatment era. Cancer Treat Rev. 2017;53:128‐137. 28110254 10.1016/j.ctrv.2016.12.006

[3] Yang JCH , Kim SW , Kim DW , et al. Osimertinib in patients with epidermal growth factor receptor mutation‐positive non‐small‐cell lung cancer and leptomeningeal metastases: the BLOOM study. J Clin Oncol. 2020;38(6):538‐547. 31809241 10.1200/JCO.19.00457 PMC7030895

[4] Hyun JW , Jeong IH , Joung A , Cho HJ , Kim SH , Kim HJ . Leptomeningeal metastasis: clinical experience of 519 cases. Eur J Cancer. 2016;56(56):107‐114. 26841095 10.1016/j.ejca.2015.12.021

[5] Ruan H , Zhou Y , Shen J , et al. Circulating tumor cell characterization of lung cancer brain metastases in the cerebrospinal fluid through single‐cell transcriptome analysis. Clin Transl Med. 2020;10(8):e246. 33377642 10.1002/ctm2.246 PMC7737787

[6] Li Y , Polyak D , Lamsam L , et al. Comprehensive RNA analysis of CSF reveals a role for CEACAM6 in lung cancer leptomeningeal metastases. NPJ Precis Oncol. 2021;5(1):90. 34625644 10.1038/s41698-021-00228-6 PMC8501028

[7] Beauchemin N , Arabzadeh A . Carcinoembryonic antigen‐related cell adhesion molecules (CEACAMs) in cancer progression and metastasis. Cancer Metastasis Rev. 2013;32(3–4):643‐671. 23903773 10.1007/s10555-013-9444-6

[8] Grunnet M , Sorensen JB . Carcinoembryonic antigen (CEA) as tumor marker in lung cancer. Lung Cancer. 2012;76(2):138‐143. 22153832 10.1016/j.lungcan.2011.11.012

[9] Singer BB , Scheffrahn I , Kammerer R , Suttorp N , Ergun S , Slevogt H . Deregulation of the CEACAM expression pattern causes undifferentiated cell growth in human lung adenocarcinoma cells. PLoS One. 2010;5(1):e8747. 20090913 10.1371/journal.pone.0008747 PMC2807459

[10] Johnson B , Mahadevan D . Emerging role and targeting of carcinoembryonic antigen‐related cell adhesion molecule 6 (CEACAM6) in human malignancies. Clin Cancer Drugs. 2015;2(2):100‐111. 27595061 10.2174/2212697X02666150602215823 PMC4997943

[11] Son SM , Yun J , Lee SH , et al. Therapeutic effect of pHLIP‐mediated CEACAM6 gene silencing in lung adenocarcinoma. Sci Rep. 2019;9(1):11607. 31474761 10.1038/s41598-019-48104-5 PMC6717735

[12] Li YS , Jiang BY , Yang JJ , et al. Leptomeningeal metastases in patients with NSCLC with EGFR mutations. J Thorac Oncol. 2016;11(11):1962‐1969. 27539328 10.1016/j.jtho.2016.06.029

[13] Liu Y , Yang S , Zhao J , et al. Cell‐free DNA from cerebrospinal fluid can be used to detect the EGFR mutation status of lung adenocarcinoma patients with central nervous system metastasis. Transl Lung Cancer Res. 2021;10(2):914‐925. 33718032 10.21037/tlcr-21-62 PMC7947414

[14] Wang Y , Luo N , Gao Y , et al. The joint detection of CEA and ctDNA in cerebrospinal fluid: an auxiliary tool for the diagnosis of leptomeningeal metastases in cancer. J Cancer Res Clin Oncol. Published online May 18, 2022. doi:10.1007/s00432-022-04053-7 PMC11797791 35583828

[15] Ferguson SD , Fomchenko EI , Guerrieri RA , Glitza Oliva IC . Challenges and advances in diagnosis and treatment of leptomeningeal disease (LMD). Front Oncol. 2021;11:800053. 35096602 10.3389/fonc.2021.800053 PMC8789647

[16] Ge M , Zhan Q , Zhang Z , et al. Different next‐generation sequencing pipelines based detection of tumor DNA in cerebrospinal fluid of lung adenocarcinoma cancer patients with leptomeningeal metastases. BMC Cancer. 2019;19(1):143. 30755180 10.1186/s12885-019-5348-3 PMC6373107

[17] Nevel KS , DiStefano N , Lin X , et al. A retrospective, quantitative assessment of disease burden in patients with leptomeningeal metastases from non‐small‐cell lung cancer. Neuro Oncol. 2020;22(5):675‐683. 32352148 10.1093/neuonc/noz208 PMC7229251

[18] Sasaki S , Yoshioka Y , Ko R , et al. Diagnostic significance of cerebrospinal fluid EGFR mutation analysis for leptomeningeal metastasis in non‐small‐cell lung cancer patients harboring an active EGFR mutation following gefitinib therapy failure. Respir Investig. 2016;54(1):14‐19. 10.1016/j.resinv.2015.07.001 26718140

[19] Kobayashi M , Miki Y , Ebina M , et al. Carcinoembryonic antigen‐related cell adhesion molecules as surrogate markers for EGFR inhibitor sensitivity in human lung adenocarcinoma. Br J Cancer. 2012;107(10):1745‐1753. 23099808 10.1038/bjc.2012.422 PMC3493859

[20] Codreanu SG , Hoeksema MD , Slebos RJC , et al. Identification of proteomic features to distinguish benign pulmonary nodules from lung adenocarcinoma. J Proteome Res. 2017;16(9):3266‐3276. 28731711 10.1021/acs.jproteome.7b00245 PMC6339813

[21] Kim EY , Cha YJ , Jeong S , Chang YS . Overexpression of CEACAM6 activates Src‐FAK signaling and inhibits anoikis, through homophilic interactions in lung adenocarcinomas. Transl Oncol. 2022;20:101402. 35358791 10.1016/j.tranon.2022.101402 PMC8968058

[22] Wu SJ , Arundhathi A , Wang HC , et al. Migration and invasion of NSCLC suppressed by the downregulation of Src/focal adhesion kinase using single, double and tetra domain anti‐ CEACAM6 antibodies. Transl Oncol. 2021;14(7):101057. 33934053 10.1016/j.tranon.2021.101057 PMC8105299

[23] Hong KP , Shin MH , Yoon S , et al. Therapeutic effect of anti CEACAM6 monoclonal antibody against lung adenocarcinoma by enhancing anoikis sensitivity. Biomaterials. 2015;67:32‐41. 26204223 10.1016/j.biomaterials.2015.07.012

[24] Walbert T , Groves MD . Known and emerging biomarkers of leptomeningeal metastasis and its response to treatment. Future Oncol. 2010;6(2):287‐297. 20146587 10.2217/fon.09.167

[25] Zhang W , Huang Y , Xu J . Cancer. In: Pan S , Tang J , eds. Clinical molecular diagnostics. Springer Singapore; 2021:261‐284.

[26] Pinon P , Wehrle‐Haller B . Integrins: versatile receptors controlling melanocyte adhesion, migration and proliferation. Pigment Cell Melanoma Res. 2011;24(2):282‐294. 21087420 10.1111/j.1755-148X.2010.00806.x

[27] Hamidi H , Ivaska J . Every step of the way: integrins in cancer progression and metastasis. Nat Rev Cancer. 2018;18(9):533‐548. 30002479 10.1038/s41568-018-0038-z PMC6629548

[28] Hoshino A , Costa‐Silva B , Shen TL , et al. Tumour exosome integrins determine organotropic metastasis. Nature. 2015;527(7578):329‐335. 26524530 10.1038/nature15756 PMC4788391

[29] Lorger M , Krueger JS , O'Neal M , Staflin K , Felding‐Habermann B . Activation of tumor cell integrin alphavbeta3 controls angiogenesis and metastatic growth in the brain. Proc Natl Acad Sci U S A. 2009;106(26):10666‐10671. 19541645 10.1073/pnas.0903035106 PMC2697113

[30] Duxbury MS , Ito H , Ashley SW , Whang EE . c‐Src‐dependent cross‐talk between CEACAM6 and alphavbeta3 integrin enhances pancreatic adenocarcinoma cell adhesion to extracellular matrix components. Biochem Biophys Res Commun. 2004;317(1):133‐141. 15047158 10.1016/j.bbrc.2004.03.018

[31] Ordonez C , Zhai AB , Camacho‐Leal P , Demarte L , Fan MM , Stanners CP . GPI‐anchored CEA family glycoproteins CEA and CEACAM6 mediate their biological effects through enhanced integrin alpha5beta1‐fibronectin interaction. J Cell Physiol. 2007;210(3):757‐765. 17167768 10.1002/jcp.20887

