# Supplementary figures and images for: CEACAM6 serves as a biomarker for leptomeningeal metastasis in lung adenocarcinoma
Source: Cancer Med. 2022 Sep 9;12(4):4521–9. doi: 10.1002/cam4.5221 (PMC9972070; doi:10.1002/cam4.5221)

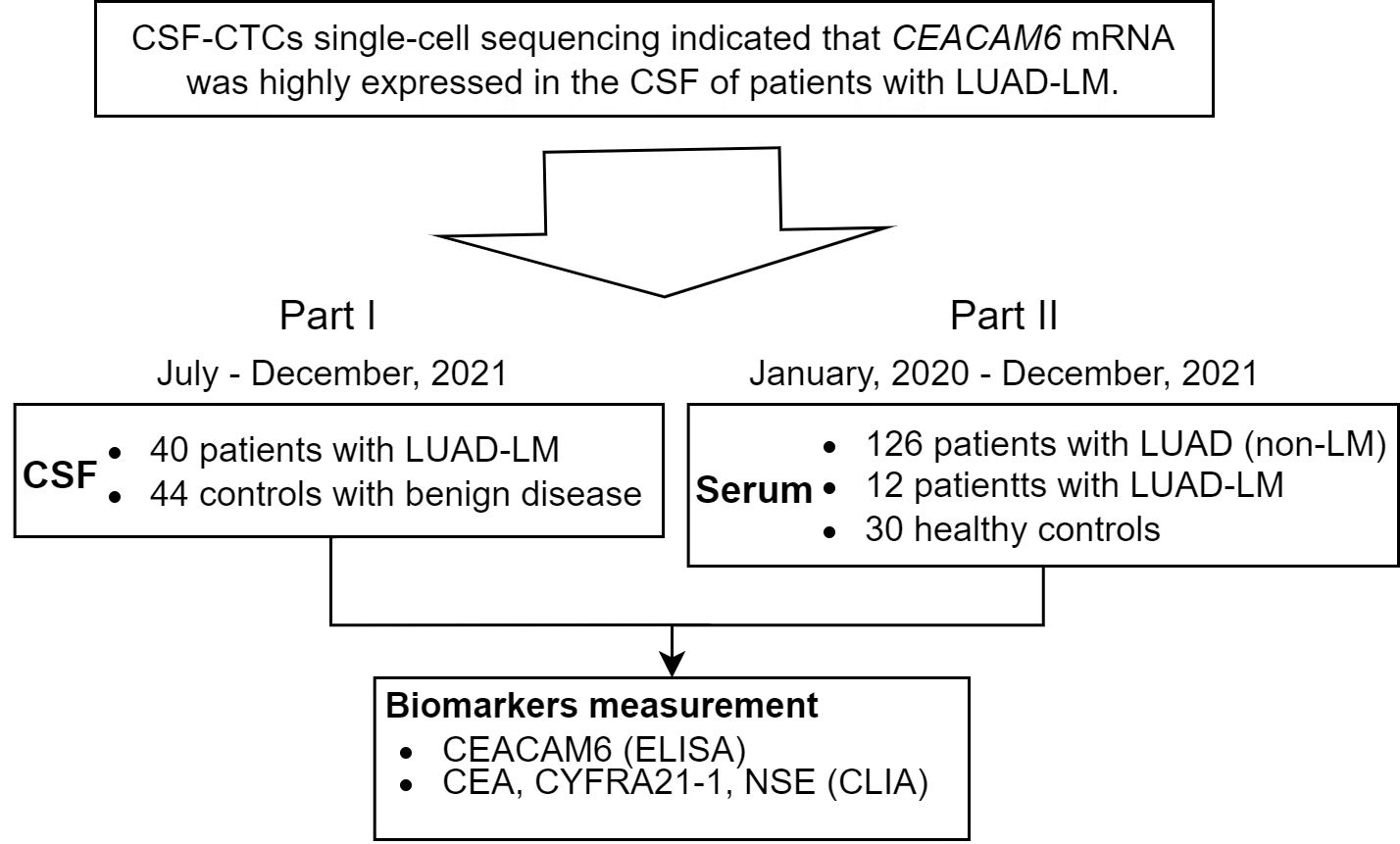

Supplement: Supplementary file 1 — Figure S1 [file CAM4-12-4521-s002.jpg]

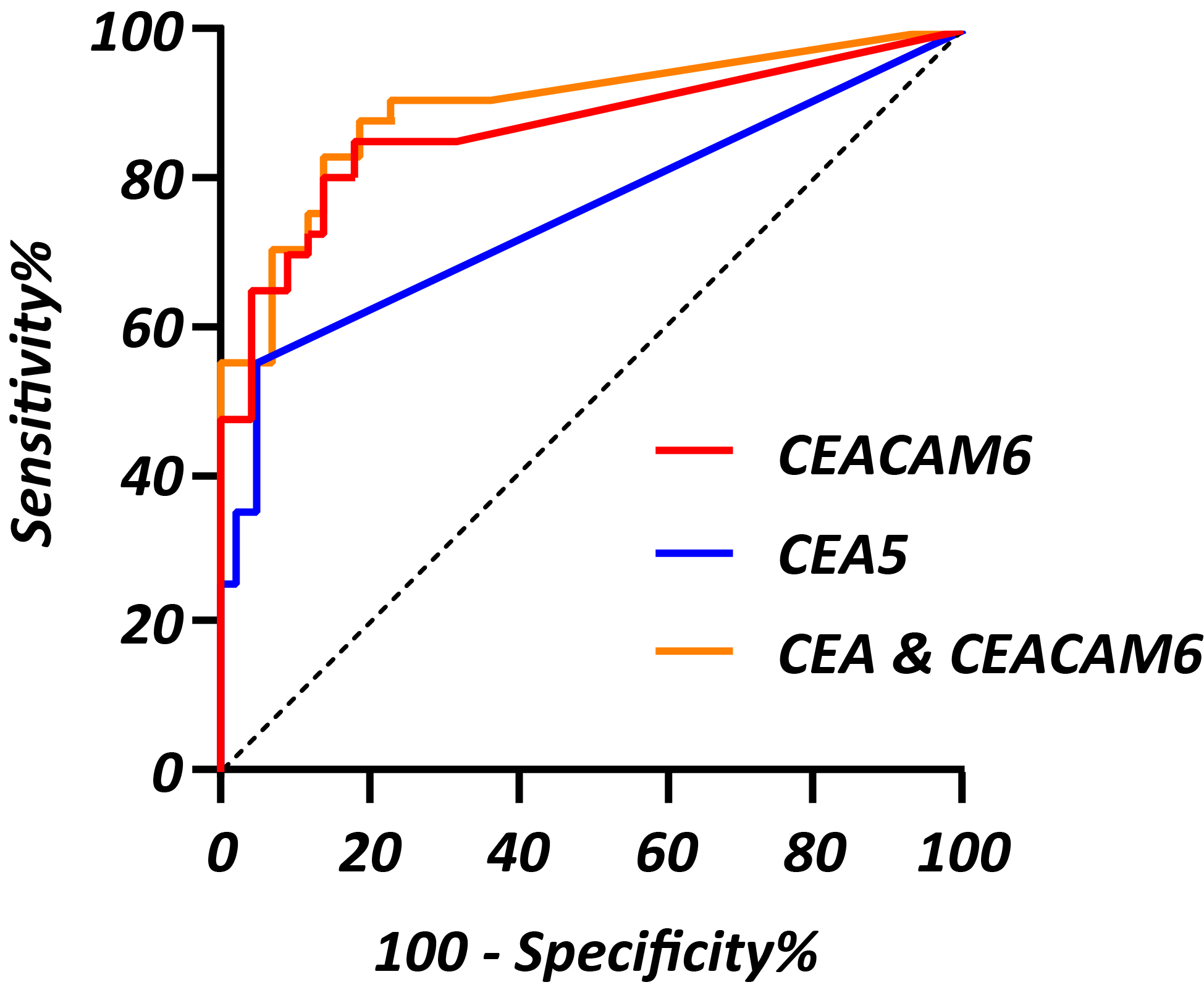

Supplement: Supplementary file 2 — Figure S2 [file CAM4-12-4521-s001.jpg]

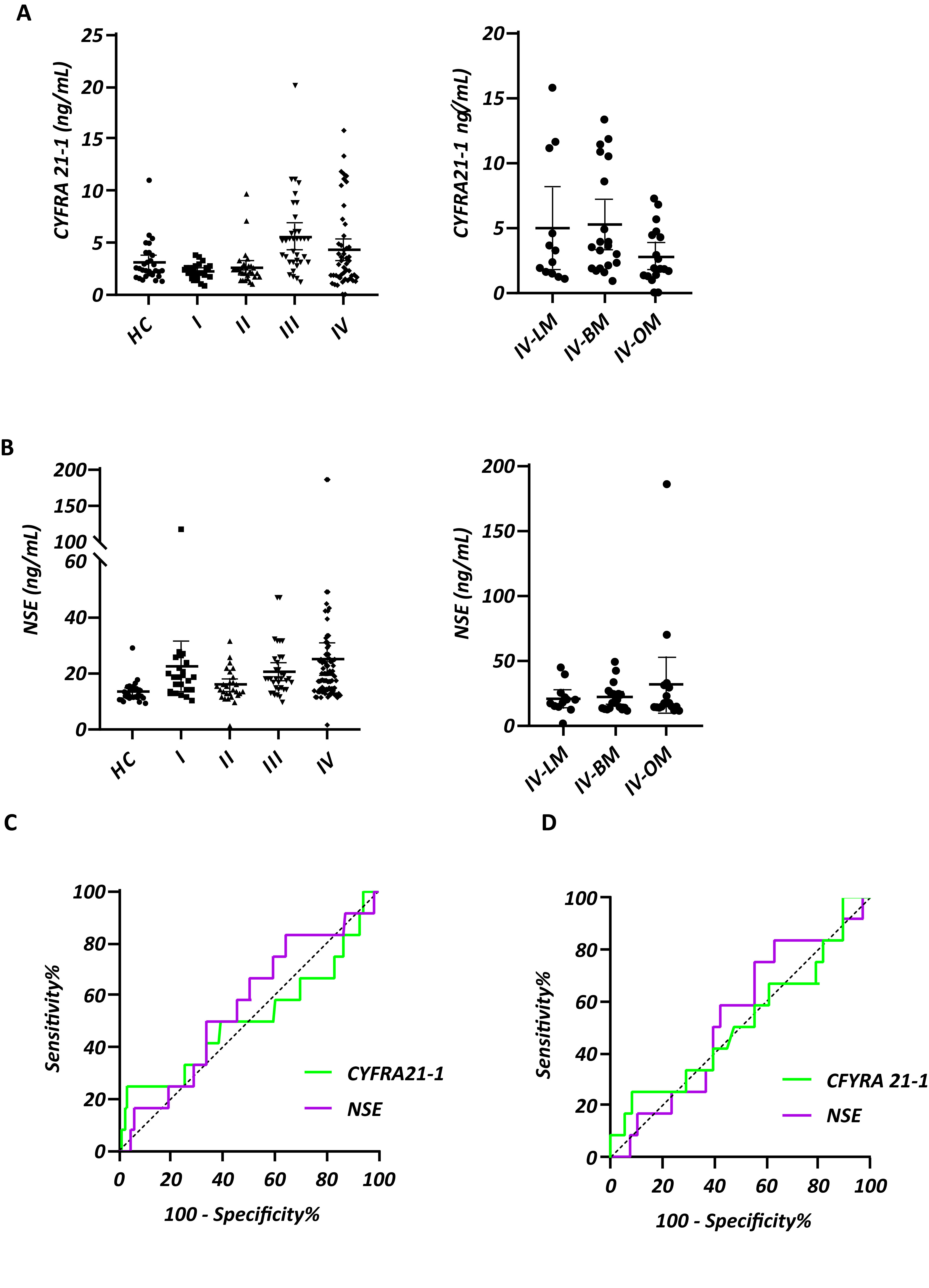

Supplement: Supplementary file 3 — Figure S3 [file CAM4-12-4521-s004.jpg]
