# Supplementary material for: CEACAM6 serves as a biomarker for leptomeningeal metastasis in lung adenocarcinoma
Source: Cancer Med. 2022 Sep 9;12(4):4521–9. doi: 10.1002/cam4.5221 (PMC9972070; doi:10.1002/cam4.5221)
Supplement: Supplementary file 4 — Table S1 Table S2 [file CAM4-12-4521-s003.docx]

**Table S1** Characteristics of patients included in CSF CEACAM6 analysis

| **Groups** | **N** | **(%)** | **Gender (Male%)** | **Age [median (25th, 75th)]** |
| --- | --- | --- | --- | --- |
| LUAD-LM | 40 |  | 42.5 | 57.0 (50.0, 65.8) |
| Normal control* | 44 |  | 52.3 | 50.0 (37.0, 58.8) |

*Referred to benign disease patients with normal results of routine CSF analysis and cytology

**Table S2** Characteristics of LUAD patients included in serum CEACAM6 analysis

| **Groups** | **N** | **(%)** | **Gender (Male%)** | **Age [median (25th, 75th)]** |
| --- | --- | --- | --- | --- |
| HC | 30 |  | 50.0 | 45.0 (38.8, 51.0) |
| LUAD | 138 |  | 55.8 | 60.0 (52.0, 67.3) |
| Stage I | 24 | (17.4) | 50.0 | 54.0 (41.3, 63.0) |
| Stage II | 29 | (21.1) | 62.1 | 58.0 (49.0, 68.5) |
| Stage III | 35 | (25.4) | 72.2 | 63.0(55.0, 69.8) |
| Stage IV | 50 | (36.2) | 44.0 | 59.0 (52.8, 68.3) |
| IV-LM | 12 | (8.7) | 50.0 | 57.5 (54.3, 61.8) |
| IV-BM | 20 | (14.5) | 40.0 | 55.5 (50.5, 66.5) |
| IV-OM | 18 | (13.0) | 44.4 | 65.5 (54.3, 69.5) |
